# The consequences of the COVID-19 pandemic on mental health and implications for clinical practice

**DOI:** 10.1192/j.eurpsy.2020.35

**Published:** 2020-04-01

**Authors:** Andrea Fiorillo, Philip Gorwood

**Affiliations:** 1 Department of Psychiatry, University of Campania “L. Vanvitelli”, Naples, Italy; 2 Editor, European Psychiatry; 3 GHU Paris Psychiatrie et Neurosciences, CMME, Hôpital Sainte-Anne, 75014 Paris, France; 4 Université de Paris, Institute of Psychiatry and Neuroscience of Paris (IPNP), INSERM U1266, 75014 Paris, France

There is a wide consensus that the COVID-19 pandemic not only affects physical health, but also mental health and well-being [1,2]. The current pandemic is changing priorities for the general population, but it is also challenging the agenda of health professionals, including that of psychiatrists and other mental health professionals [3]. Everywhere in the world, psychiatric clinics are modifying their practice in order to guarantee care and support to persons with mental health problems, but also to those who are not mentally ill and are suffering from the psychosocial consequences of the pandemic. The number of those who will need psychiatric help is going to increase in the next weeks or months, requiring a reconsideration of our current practices. From a psychopathological viewpoint, the current pandemic is a relatively new form of stressor or trauma for mental health professionals [4]. It has been compared with natural disasters, such as earthquakes or tsunamis [5]. But in those cases, the emergencies are usually localized, limited to a specific area and to a given time; people know that they can escape, if they want to or if they have the possibility to do so [6]. It has also been compared with wars and international mass conflicts. But in those circumstances, the enemy is easily recognizable, while in pandemic the “threat” can be everywhere and it can be carried by the person next to us [7].

We consider that the mental health and psychosocial consequences of the COVID-19 pandemic may be particularly serious for at least four groups of people: (a) those who have been directly or indirectly in contact with the virus; (b) those who are already vulnerable to biological or psychosocial stressors (including people affected by mental health problems); (c) health professionals (because of higher level of exposure); and (d) even people who are following the news through numerous media channels.

The pandemic and the related containment measures—namely quarantine, social distancing, and self-isolation—can have a detrimental impact on mental health. In particular, the increased loneliness and reduced social interactions are well-known risk factors for several mental disorders, including schizophrenia and major depression. Concerns about one’s own health and that of their beloved ones (particularly elderly or suffering from any physical illness), as well as uncertainty about the future, can generate or exacerbate fear, depression, and anxiety. If these concerns are prolonged, they may increase the risk of serious and disabling mental health conditions among adult males and females, including anxious disorders including panic, obsessive–compulsive, stress, and trauma-related disorders. A group at a particularly high risk is represented by infected people, physicians, and nurses working in emergency units and resuscitation departments. It is likely that in the next months—when the pandemic is over—we may have a shortage of health professionals due to burnout and mental exhaustion [8]. Another aspect which should be considered is related to stigma and discrimination toward infected people and their family members. Fighting social stigma toward those treating and caring for people with COVID-19 should be another priority for mental health professionals in the next months. Finally, Internet is spreading very rapidly a large amount of uncontrolled news. This information overload has been defined “infodemic,” with the risk of fake news running faster than the virus itself, and creating uncertainties and worries. This should be regulated by a continuous interaction with media and also by national regulations. Another consequence of the pandemic on mental health practice may be that psychiatric problems will be considered less important than physical ones. We should continue to advocate for our patients and their caregivers; our patients often need long-term treatment, continuous support and advices, personal meetings with their physicians or therapists. Their rights to be treated, also in a period of social distancing, should be preserved even though mental health services may be overloaded by a considerable number of requests for psychiatric consultations.

Many of these psychosocial and mental health consequences of the pandemic will have to be addressed by psychiatrists and mental health professionals in the months to come. Most probably we will face an increase of mental health problems, behavioral disturbances, and substance-use disorders, as extreme stressors may exacerbate or induce psychiatric problems. In order to reduce the risk of developing mental health problems, simple advices may be provided to the general population:

1. Limit the sources of stress: to rely on a limited amount of official information sources only and to limit the time of the day devoted to this activity, disregarding those which come from unofficial channels and uncontrolled sources.
2. Break the isolation: to increase the communication with friends, family members, and loved ones, even if at a distance. Video-chat or group calls with family members may help to reduce loneliness and precariousness. In case of insufficient social network, professional helplines are particularly useful, if managed by qualified trained professionals.
3. Maintain your usual rhythm: keep a regular routine, by having regular sleep–wake rhythms and diet patterns. Addictive behaviors might be particularly at risk of rebound or relapses, therefore intellectual, physical, and social (even if virtual) activities will be useful.
4. Focus on the benefit of the isolation: we should indeed be conscious that this is a transient period and that this isolated time is needed as we are not only saving our health, but also protecting all others by stopping the epidemic, and therefore shaping our own future.
5. Ask for professional help: getting a psychiatric help or consultation, if the effects of stress is becoming too invasive, is always possible, even if with different modalities. Almost all psychiatric clinics are now equipped for providing support, emotional defusing, problem-solving strategies, and psychiatric consultations—also at a distance.

The pandemic will be over, but its effects on mental health and well-being of the general population, health professionals, and vulnerable people will remain for a long time. We hope that all of the mental health community will have very quickly the opportunity to take care of patients in more conventional and personalized ways. Crises also reveal resilience skills and quality of links, the solidarity observed between European countries for severe cases (exchanging patients, material, and competencies) is a nice example to follow.
